# Marine biofilms as reservoirs of antimicrobial bacteria: molecular identification and functional characterization of epibiotic culturable communities from biofouling invertebrates in northern Tunisia

**DOI:** 10.3389/fmicb.2026.1802528

**Published:** 2026-06-29

**Authors:** Yosra Alouadi, Bilel Hassen, Imen Jaouani, Radhia Mraouna, Jamila Ben Souissi, Monia El Bour

**Affiliations:** 1 Group of Aquatic Bacteriology and Biotechnology (GAB2), LR16INSTM05, The National Institute of Science and Technology of the Sea, University of Carthage, Salammbô, Tunisia; 2 National Institute of Agronomy of Tunisia (INAT), Biodiversity, Biotechnology and Climate Change Laboratory (LR11ES09), University Tunis El Manar, Tunis, Tunisia

**Keywords:** antibiotic resistance, antimicrobial activity, biofouling, DNA barcoding, epibiotic culturable bacteria, exoenzymes, Marina in northern Tunisia

## Abstract

Marine biofouling communities constitute a rich reservoir of microbial diversity and represent a promising source of bioactive metabolites. In this study, we investigated culturable epibiotic bacteria associated with fouling invertebrates from the Marina in northern Tunisia, with a focus on their enzymatic activities, antimicrobial potential, and antibiotic resistance profiles. A total of 52 bacterial isolates were recovered from 23 fouling invertebrate hosts and characterized using DNA barcoding and molecular identification. The epibiotic culturable bacterial community was dominated by members of the genera *Vibrio*, *Photobacterium*, *Halomonas*, and *Pseudomonas*. Enzymatic screening revealed a high hydrolytic potential, with DNase (71.2%), lipase (65.4%), and gelatinase (59.6%) being the most prevalent activities. Antimicrobial assays showed that a substantial proportion of isolates exhibited inhibitory activity against at least one pathogenic indicator strain, whereas antibiotic susceptibility testing revealed frequent resistance, particularly to fosfomycin and cefoxitin. Together, these findings highlight the dual nature of epibiotic culturable bacteria in the Marina in northern Tunisia, acting both as a reservoir of biotechnologically valuable antimicrobial producers and as potential carriers of antibiotic resistance, underscoring their ecological relevance and public health significance in Mediterranean coastal ecosystems.

## 1 Introduction

Microbial biofilms are ubiquitous in marine environments and represent one of the most successful microbial life strategies in aquatic ecosystems. Biofilms are defined as structured communities of microorganisms, predominantly bacteria, that adhere to biotic or abiotic surfaces and are embedded in a self-produced extracellular polymeric substance (EPS) matrix composed mainly of polysaccharides, proteins, extracellular DNA, and water. This organization confers important adaptive advantages over planktonic lifestyles, including increased tolerance to hydrodynamic stress, environmental fluctuations, antimicrobial compounds, and nutrient limitations (Costerton et al., 1995; Flemming et al., 2016). Biofilm formation is a dynamic and multistep process involving initial reversible attachment, irreversible adhesion, microcolony development, EPS production, and maturation into complex three-dimensional structures with internal nutrient and waste transport channels (Parsek and Singh, 2003; Sauer et al., 2022).

In marine ecosystems, biofilms rapidly colonize all immersed surfaces, including natural substrates such as rocks, sediments, and living organisms, as well as artificial structures such as ship hulls, pontoons, aquaculture facilities, and port infrastructures. These microbial assemblages play a fundamental ecological role by contributing to biogeochemical cycling, organic matter degradation, and microbial food webs. However, marine biofilms are also implicated in deleterious processes such as microbiologically influenced corrosion and marine biofouling, with significant ecological and economic implications (Videla and Characklis, 1992; Decho, 2000; Dang and Lovell, 2016).

Marine biofouling is built by the accumulation of microorganisms, algae, and sessile invertebrates on submerged surfaces and is a widespread phenomenon in coastal environments worldwide (Catão et al., 2019; Gomez-Banderas, 2022; Abid et al., 2024). Biofouling alters benthic–pelagic coupling, facilitates the dispersal of invasive and pathogenic species, and generates substantial economic losses due to increased hydrodynamic drag, corrosion, and maintenance costs of maritime infrastructures (Qian et al., 2007; Antunes et al., 2013). At first, the biofouling began by a film or microbial biofilms (microfouling), followed by the settlement of algae and macrofouling organisms such as barnacles, bryozoans, mussels, and polychaetas.

Microbial biofilms are recognized as the main drivers of biofouling dynamics. Early-stage bacterial biofilms modify the physicochemical properties of surfaces and produce chemical cues that influence larval settlement and community succession. Consequently, the composition and functional traits of biofilm-forming bacterial communities play a decisive role in determining the structure, persistence, and intensity of fouling assemblages (Camps et al., 2014; Romeu and Mergulhão, 2023; Shimeta et al., 2023). Investigation of the biofilm-associated bacteria is therefore essential for elucidating biofouling mechanisms and developing sustainable antifouling strategies.

Beyond artificial substrates, biofilms also form extensively on living marine organisms, giving rise to complex epibiotic microbial communities. Marine invertebrates as sponges, corals, mollusks, ascidians, bryozoans, and polychaetas host diverse bacterial assemblages on their external surfaces. These epibiotic biofilms are shaped by host-specific characteristics, surface chemistry, environmental conditions, and microbial interactions, and constitute a key ecological interface between the host organism and the surrounding marine environment (Wahl et al., 2012).

Epibiotic bacteria associated with marine invertebrates are increasingly recognized for their ecological and functional versatility. Many of these bacteria produce bioactive secondary metabolites, including antimicrobial, antibiofilm, and antifouling compounds, which may inhibit the settlement of competing microorganisms and macrofoulers, thereby contributing to host defense and surface homeostasis (Qian et al., 2007). In addition, epibiotic bacterial communities commonly secrete extracellular hydrolytic enzymes such as lipases, DNases, proteases, cellulases, chitinases, and amylases. These enzymes play key roles in organic matter degradation, biofilm remodeling, and nutrient cycling, while also representing valuable resources for biotechnological applications, including pharmaceuticals, industrial bioprocesses, and environmentally friendly antifouling technologies (Doghri et al., 2015; Lavrador et al., 2024; Migaou et al., 2024).

Marine biofilms are also increasingly recognized as hotspots for the emergence and dissemination of antibiotic resistance. Coastal environments are subjected to strong selective pressures resulting from anthropogenic activities such as wastewater discharge, agricultural runoff, industrial effluents, and intense maritime traffic. These pressures may promote the selection and persistence of antibiotic-resistant bacteria within biofouling and epibiotic communities, raising concerns regarding their potential role as environmental reservoirs of resistance genes and their implications for ecosystem and public health (Jebara et al., 2022; Hassen et al., 2025).

Despite their ecological and biotechnological relevance, biofouling-associated bacterial communities in the Southern Mediterranean, particularly along North African coastlines, remain poorly investigated. In Tunisia, available data were on the enzymatic, antimicrobial, and antifouling potential of bacteria associated with marine fouling algal assemblages (Qian et al., 2022; Cherif et al., 2024).

The present study, represent the first work as a comprehensive investigation of culturable epibiotic bacteria associated with marine invertebrates in marina’s area in northern Tunisia, in aim to characterize the macrofaunal community using DNA barcoding, identify bacterial communities associated with fouling invertebrates, and evaluate the biochemical and antimicrobial activities of the isolates against human and environmental pathogens, determine their antibiotic resistance and susceptibility profiles, and confirm the taxonomic identity of bacterial isolates using the 16S rRNA gene sequencing. The findings, integrating macrofaunal composition with bacterial functional traits, enzymatic activity, and antimicrobial potential, provide novel insights into the ecological and biotechnological significance of epibiotic culturable bacteria in the Marina in northern Tunisia and offer valuable information about biofilm-mediated interactions, exploring bacterial resources for antifouling applications, and assessing the environmental health risks associated with antibiotic-resistant bacteria in coastal waters.

## 2 Materials and methods

### 2.1 Sample collection and pre-processing

**Figure 1:**
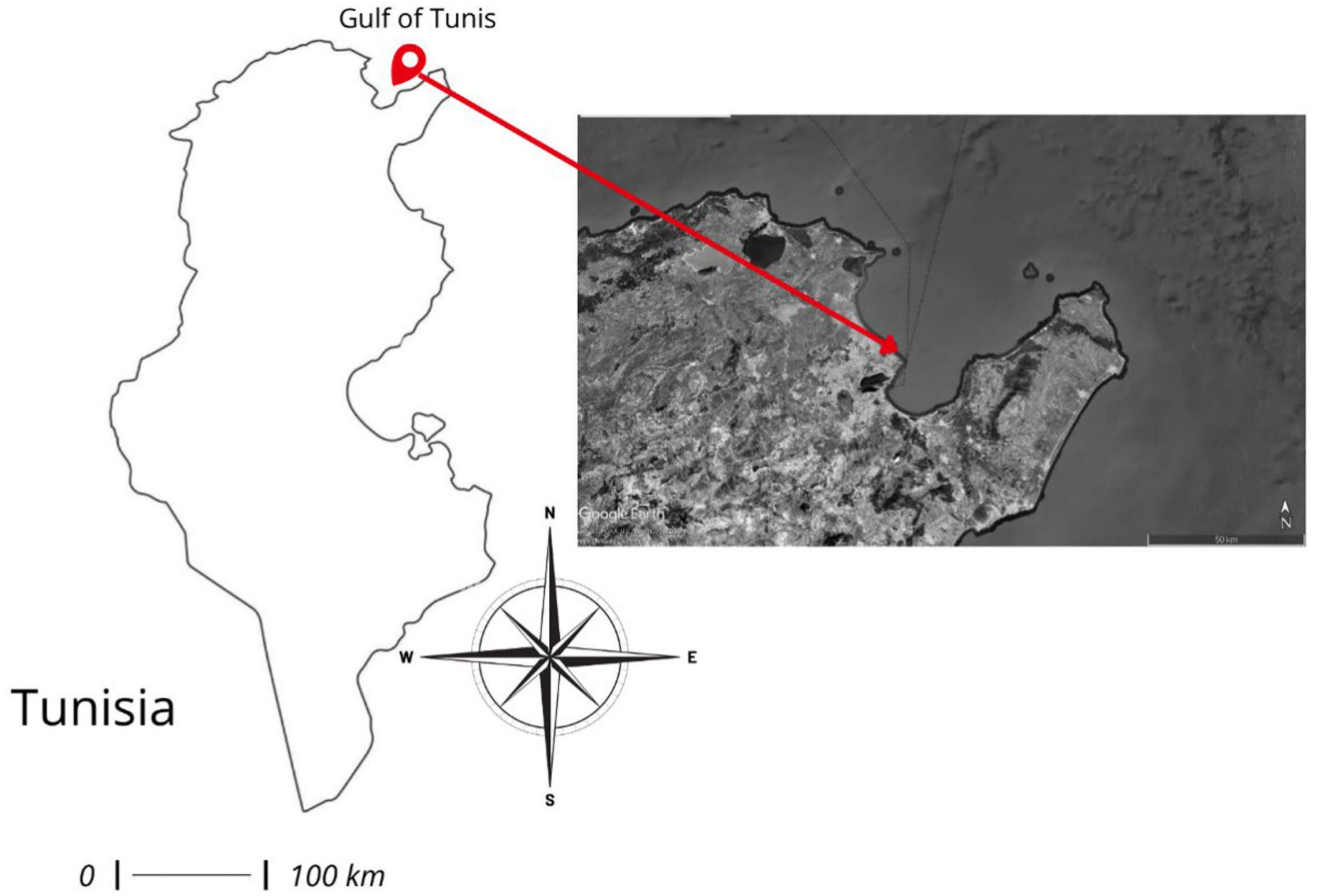
Sampling location in the Marina in northern Tunisia. Map indicating the sampling site at the Marina, northern Tunisia, where fouling invertebrate specimens and associated biofilm were collected from artificial submerged structures (concrete quay walls, vessel hulls, and mooring lines).

Field sampling was carried out in November 2024 at the Marina in northern Tunisia (Figure 1). The sampling effort focused on submerged biofouling communities developing on diverse substrates, including synthetic fiber mooring lines, vessel hulls, and concrete quay walls situated at the water–air interface. Environmental parameters of seawater were measured *in situ* in the Marina in northern Tunisia during the sampling campaign to characterize the physicochemical conditions influencing biofilm development. Seawater temperature was continuously recorded using a HOBO MX2201 data logger, while salinity was measured at 1.5 m depth using a WTW conductivity meter equipped with a TetraCon^®^ probe. The pH was determined using a WTW pH meter with a dedicated electrode, and dissolved oxygen was measured using an FDO optical probe connected to a multiparameter WTW instrument. In addition, site characteristics including depth, water transparency, and geographical coordinates were recorded. Collected invertebrate specimens were immediately preserved in 95% ethanol for subsequent morphological identification and DNA barcoding.

At the same time, biofilm material was aseptically scraped from both the surfaces of the organisms and the adjacent substrates using sterile scalpels and transferred into pre-sterilized containers. All samples were kept at 4 °C during transport to the laboratory to preserve microbial viability and integrity prior to bacteriological isolation, phenotypic characterization, and molecular analyses.

### 2.2 DNA extraction and amplification of the COI barcode region for species identification

Following tissue sampling, genomic DNA was isolated from 10 to 20 mg of material using the Pure Link™ Genomic DNA Mini Kit (Thermo Fisher Scientific) following the manufacturer’s protocol. A fragment of the mitochondrial Cytochrome C Oxidase I (COI) gene, selected for its high evolutionary rate and utility as a species-specific barcode, was then targeted for amplification via polymerase chain reaction (PCR) using the universal primer LCO1490 (5′-GGTCAACAAATCATAAAGATATTGG-3′) and HCO2198 (5′-TAAACTTCAGGGTGACCAAAAAATCA-3′) (Folmer et al., 1994).

### 2.3 Evolutionary relationships of taxa for identified species

The evolutionary relationships among taxa were inferred using the Neighbor-Joining method. Node support was assessed by bootstrap analysis with 1,000 replicates, and bootstrap values are indicated at the corresponding nodes. The phylogenetic tree was constructed to scale, with branch lengths proportional to evolutionary distances. These distances were calculated using the Maximum Composite Likelihood method and are expressed as the number of base substitutions per site. For each internal node, the proportion of sites containing at least one unambiguous nucleotide in at least one sequence of each descendant clade was reported. The analysis included 11 nucleotide sequences. Ambiguous positions were excluded using the pairwise deletion option, resulting in a final alignment of 668 positions. All phylogenetic analyses were performed using MEGA version 11.

### 2.4 Preparation of homogenate and bacterial isolation

In the laboratory, biofilm material and invertebrate tissues were aseptically transferred into sterile tubes containing 9 mL of physiological saline and homogenized to release associated microbes. A 100 μL aliquot of each homogenate was streaked onto Tryptic Soy Agar (TSA) supplemented with 2% NaCl, and Thiosulfate Citrate Bile Salts Sucrose (TCBS) agar. Plates were incubated at 30 °C for 48 h. Pure isolates were obtained through repeated streaking and subsequently cultured in Tryptic Soy Broth (TSB). Long-term storage was performed in Nutrient Broth with 50% glycerol at −80 °C.

### 2.5 Preliminary characterization

All bacterial isolates were subjected to standard microbiological characterization. Catalase and oxidase activities were determined according to Prescott et al. (2003) to evaluate the respiratory metabolism of each isolate. Gram staining was performed to determine cell wall type, and microscopic observations were conducted to assess cellular morphology and arrangement.

### 2.6 Bacterial strain identification and genomic analysis

Bacterial genomic DNA was extracted using the boiling method. For strain differentiation at the species and inter-species level, the 16S-23S intergenic spacer (ITS) region was amplified by PCR using the primers ITS-F (5′-GTCGTAACAAGGTAGCCGTA-3′) and ITS-R (5′-CTACGGCTACCTTGTTACGA-3′), as described by Hassen et al. (2018). The amplification products were resolved on a 1% agarose gel stained with SYBR Green (Atlas Clear-Sight DNA Stain, Bio-Atlas, Istanbul, Türkiye) and visualized under UV light. The resulting ITS profiles were compared based on banding patterns, and isolates sharing identical profiles were grouped into ITS genotypes. The distribution of these genotypes across different host organisms was further examined to assess the occurrence of similar bacterial taxa among hosts. A representative isolate from each distinct ITS genotype was then selected for further identification by partial 16S rRNA gene sequencing. The 16S rRNA gene was amplified using the universal primers F27 (5′-AGAGTTTGATCCTGGCTCAG-3′) and R1492 (5′-TACGGCTACCTTGTTACGACTT-3′). The resulting sequences were aligned and compared using the BLAST algorithm on the NCBI database.^1^

### 2.7 Phylogenetic dendrogram for bacterial strains

Phylogenetic relationships were inferred using the Maximum Likelihood (ML) method. The robustness of the inferred topology was evaluated through bootstrap analysis with 1,000 replicates, and the resulting bootstrap consensus tree was used to illustrate the evolutionary relationships among the analyzed taxa. Branches supported by less than 50% bootstrap replicates collapsed, and bootstrap support values (expressed as percentages) were indicated at the corresponding nodes. The initial tree for the heuristic search was selected by comparing the log-likelihood values of Neighbor-Joining (NJ) and Maximum Parsimony (MP) trees, with the topology presenting the highest likelihood retained as the starting tree. The NJ tree was generated using a matrix of pairwise distances calculated with the p-distance method, while the MP tree corresponded to the shortest tree obtained from 10 independent searches using randomly generated starting trees. Reference sequences used in the phylogenetic analysis were selected based on their highest sequence similarity to the obtained isolates, as determined by BLAST analysis. Whenever possible, sequences from type strains were preferentially included. The nomenclatural status of the selected strains was verified using the List of Prokaryotic names with Standing in Nomenclature database to ensure the use of valid and up-to-date taxonomy. The final dataset included 38 nucleotide sequences with 1,567 aligned positions. All evolutionary analyses were conducted using MEGA version 12.

### 2.8 Antimicrobial activity assay

The antibacterial activity of the bacterial isolates was assessed using an agar spot diffusion assay against a panel of well-characterized reference strains. This panel comprised internationally recognized strains from the American Type Culture Collection (ATCC, Manassas, VA, USA), including *Escherichia coli* ATCC 14948, *Staphylococcus aureus* ATCC 25923, *Vibrio anguillarum* ATCC 12964ᵀ, *Vibrio alginolyticus* ATCC 17749ᵀ, *Pseudomonas aeruginosa* ATCC 27853, *Listeria monocytogenes* ATCC 7644, and *Candida albicans* ATCC 10231. Additionally, *Salmonella typhi* C52, *Salmonella* spp., *Paenibacillus larvae*, and *Pseudomonas fluorescens* AH2 (Danish Institute for Fisheries Research, Denmark) were included to represent clinically relevant human pathogens, foodborne bacteria, and aquaculture-associated microorganisms. The use of these standardized reference strains allows for reproducible and comparable evaluation of the antimicrobial potential of isolates. Briefly, indicator strains were cultured in nutrient broth for 24 h at 30 °C, and suspensions were adjusted to a 0.5 McFarland standard in sterile physiological saline water. A volume of 100 μL of each standardized suspension was spread evenly onto Mueller-Hinton Agar (MHA) plates. Test isolates were grown in Tryptic Soy Broth (TSB) at 28 °C for 24 h. Subsequently, 10 μL aliquots of each test culture were spotted onto the inoculated MHA plates. After incubation at 30 °C for 24 h, antimicrobial activity was recorded as the presence of a clear inhibition zone surrounding the spot. All assays were performed in triplicate (Cherif et al., 2024).

### 2.9 Biochemical and enzymatic profiling of bacterial isolates

The biochemical potential of the bacterial isolates was characterized through a series of qualitative enzymatic assays performed in triplicate. Lipase activity was assessed on Tween 80-supplemented agar, with positive activity indicated by an opaque halo formation after 24 h at 37 °C. DNase production was evaluated using toluidine blue reagent, where a pink halo around colonies after 48 h at 28 °C confirmed DNA degradation capability. Lecithinase activity was detected through opaque zone formation on egg yolk emulsion agar after 24–72 h at 30 °C. The ability to utilize complex carbon sources was examined through starch hydrolysis (amylase) visualized by clear zones after Lugol’s iodine application, gelatin liquefaction (gelatinase) detected using mercury chloride solution, and cellulose degradation (cellulase) revealed by Congo red staining. Chitinase production, reflecting the capacity to degrade this abundant marine polysaccharide, was identified by clearance zones on chitin-supplemented agar. Hemolytic activity was determined by the presence of colorless zones on horse blood agar after 48–72 h at 30 °C. All methodological procedures were adapted from established protocols (Cherif et al., 2024; Pinky and Sheila, 2018; Satpute et al., 2008).

### 2.10 Antibiotic susceptibility testing

The antibiotic susceptibility of the bacterial isolates was evaluated using the disk diffusion method on Mueller–Hinton agar (MHA), according to the recommendations of the Antibiogram Committee of the French Society of Microbiology (CA-SFM, 2025). Standard antibiotic disks were used with the following concentrations: cefoxitin (5 μg), ceftazidime (30 μg), tobramycin (10 μg), chloramphenicol (30 μg), ticarcillin (75 μg), tetracycline (30 μg), ceftriaxone (30 μg), fosfomycin (50 μg), aztreonam (30 μg), sulfonamide (300 μg), and levofloxacin (5 μg).

This antibiotic panel was selected based on its clinical relevance and frequent application in human and aquaculture settings, as previously described by Pastor (2002) and Romanenko et al. (2005). Plates were incubated at 30 °C for 24 h, after which the diameters of inhibition zones surrounding each antibiotic disk were measured in millimeters. Results were interpreted according to the European Committee on Antimicrobial Susceptibility Testing (EUCAST) Clinical Breakpoint Tables version 16.0 (valid from 01 January 2026) to determine the resistance or susceptibility profile of each isolate. Although these breakpoints are primarily established for clinical isolates, they were applied here as a reference framework due to the lack of standardized criteria for environmental bacteria. Therefore, the resistance profiles reported should be interpreted as indicative of reduced susceptibility patterns rather than direct predictors of clinical treatment outcomes.

### 2.11 Statistical analysis of bacterial diversity and functional traits

Bacterial morphotype diversity among the 51 isolates was estimated using three complementary indices: the Shannon–Wiener diversity index (H′), the Simpson diversity index (D), and the Pielou evenness index (E), calculated from the relative abundance of the five identified morphotypes. The Multiple Antibiotic Resistance (MAR) index was calculated for each isolate using the formula MAR = a/b, where a represents the number of antibiotics to which an isolate was resistant and b represents the total number of antibiotics tested (*b* = 12), following the method described by Krumperman (1983). A MAR index value exceeding 0.2 is considered indicative of origin from a high antibiotic selection pressure environment. Antibiotic susceptibility testing could only be performed on 47 of the 52 isolates, as five isolates failed to exhibit sufficient growth under the experimental conditions and were therefore excluded from MAR index calculations. The relationship between enzymatic activity and antibiotic resistance, and between enzymatic activity and antimicrobial activity against indicator strains, was assessed using Spearman’s rank correlation test applied to all successfully tested isolates. Statistical significance was set at *p* < 0.05. All calculations were performed using Microsoft Excel (Microsoft 365, Microsoft Corp., Redmond, WA, USA). Shannon, Simpson, and Pielou indices were computed using standard arithmetic formulas. Spearman’s rank correlation coefficients were calculated using the RANK. AVG function to handle tied ranks, followed by manual computation of the correlation coefficient. MAR indices were calculated using cell-based arithmetic operations.

## 3 Results

### 3.1 Taxonomic identification of fouling organisms

**Figure 2:**
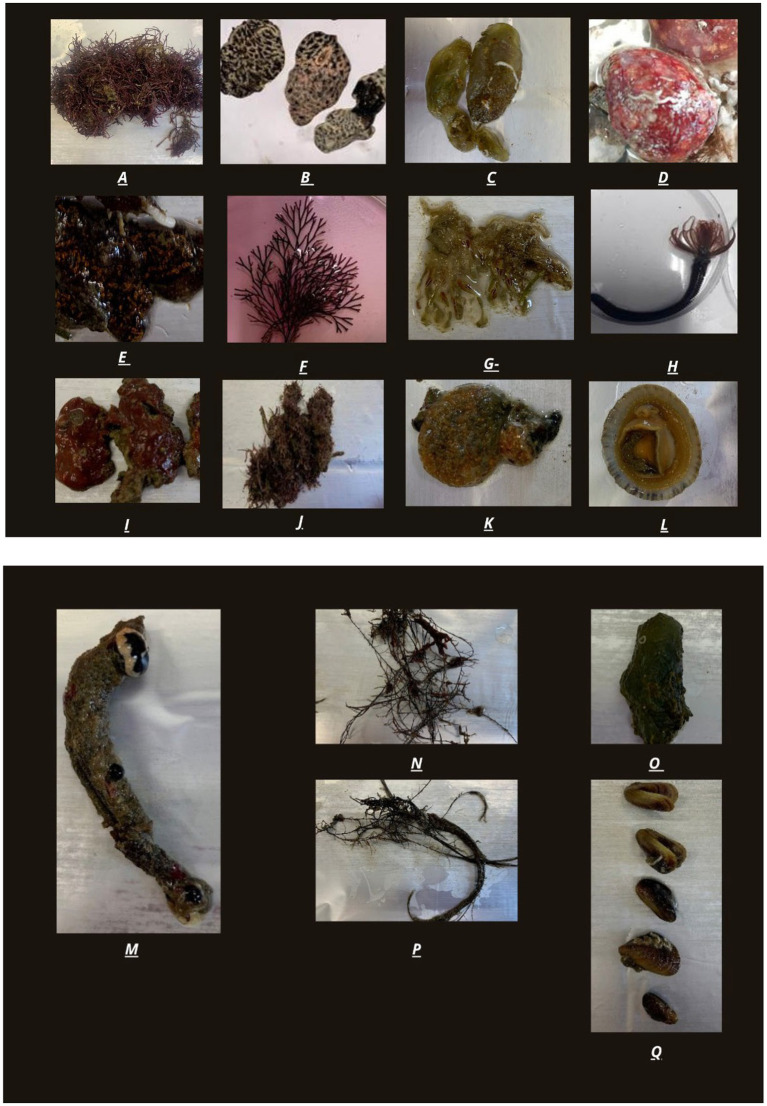
Representative marine invertebrates and macroalgae associated with biofilms collected from a marina in northern Tunisia. **(A)** *Corallina caespitosa*, **(B)** *Distaplia bermudensis*, **(C)** *Ciona intestinalis*, **(D)** *Actinia fragacea*, **(E)** *Botrylloides niger*, **(F)** *Amphiroa rigida*, **(G)** *Ecteinascidia turbinata*, **(H)** *Branchiomma luctuosum*, **(I)** *Amphiroa beauvoisii*, **(J)** *Chondrus crispus*, **(K)** *Pectinaria magnifica*, **(L)** *Patella caerulea*, **(M)** *Teredo navalis*, **(N)** *Kirchenpaueria pinnata*, **(O)** *Microcosmus squamiger*, **(P)** *Sargassum muticum*, and **(Q)** *Brachidontes pharaonis*. Images are not presented to scale. Specimen sizes ranged approximately from 10 mm to 5 cm.

DNA barcoding combined with morphological identification revealed the presence of taxonomically diverse fouling organisms associated with marine biofilms (Figure 2). Phylogenetic reconstruction based on COI sequences resolved the sampled taxa into well-supported clades corresponding to major marine lineages, consistent with their morphological assignment. The dataset encompassed representatives of macroalgae, tunicates (Ascidiacea), bryozoans, mollusks, cnidarians, annelids, hydrozoans, and shipworms (Table 1).

**Table 1:** Taxonomic composition of the biofouling macrofauna in the Marina in northern Tunisia.

Major group	Identified species	Sampling substrate (Marina)	Main reference(s)
Macroalgae (Rhodophyta and Phaeophyceae)	*Chondrus crispus, Corallina caespitosa, Amphiroa rigida, Amphiroa beauvoisii, Sargassum muticum*	Mooring lines; quay walls	Gopu and Selvam (2020)
Tunicates (Ascidiacea)	*Distaplia bermudensis, Microcosmus squamiger, Ecteinascidia turbinata, Botrylloides niger, Ciona intestinalis*	Vessel hulls; mooring lines; quay walls	MacIver et al. (2017)
Bryozoans	*Pectinella magnifica*	Mooring lines; quay walls	Reverter-Gil and Souto (2019)
Cnidarians (Anthozoa)	*Actinia fragacea*	Quay walls (water–air interface)	Pereira et al. (2021)
Mollusks (Gastropoda)	*Patella caerulea*	Quay walls (water–air interface)	Zaâbi et al. (2010)
Bivalves (Shipworms)	*Teredo navalis, Brachidontes pharaonis*	Vessel hulls; mooring lines	Zenetos et al. (2005)
Annelids (Polychaeta)	*Branchiomma luctuosum*	Mooring lines; vessel hulls	Zenetos et al. (2005)
Hydrozoans (Cnidaria)	*Kirchenpaueria pinnata*	*Mooring lines; quay walls*	Peña Cantero and García Carrascosa (2002)

Species identified through integrative DNA barcoding (COI gene) and morphological examination of specimens collected from submerged artificial structures.

**Figure 3:**
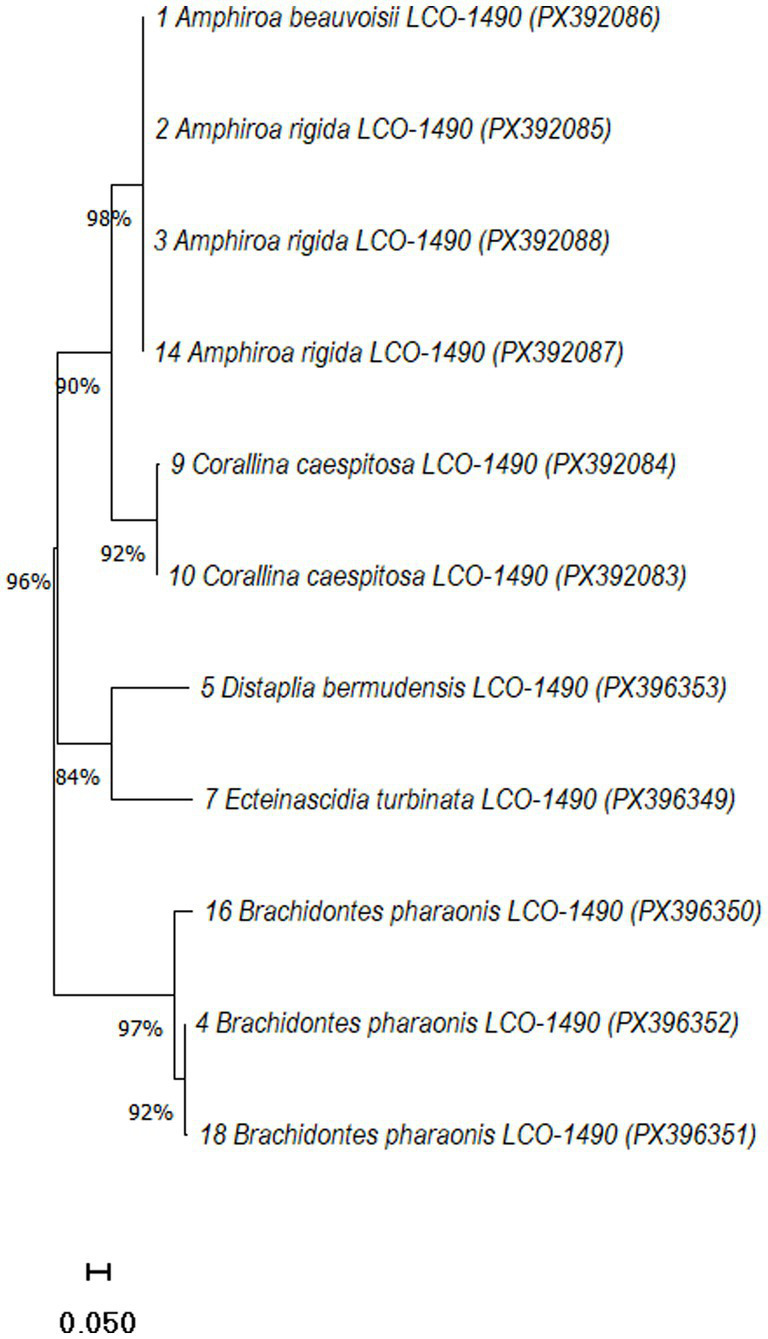
Neighbor-Joining phylogenetic tree based on mitochondrial cytochrome c oxidase subunit I (COI) gene sequences showing the evolutionary relationships among fouling invertebrate and algal species collected in this study. The tree was inferred using the Neighbor-Joining method with bootstrap values (%) based on 1,000 replicates indicated at the nodes. Evolutionary distances were computed using the Maximum Composite Likelihood method and are expressed as the number of base substitutions per site. The analysis included 11 nucleotide sequences; ambiguous positions were removed using the pairwise deletion option, resulting in a final dataset of 668 positions. The scale bar represents 0.05 substitutions per site. Numbers preceding taxon names correspond to specimen identifiers. All sequences were generated in this study and deposited in GenBank; accession numbers are given in parentheses. Analyses were performed using MEGA11.

Macroalgal taxa formed distinct and strongly supported clades, with red algae clustering into separate lineages represented by *Chondrus crispus*, *Corallina caespitosa*, *Amphiroa rigida*, and *Amphiroa beauvoisii*. The latter two species grouped closely within the Corallinaceae, confirming their phylogenetic affinity (Figure 3). The brown alga *Sargassum muticum* formed a separate lineage, reflecting its distant evolutionary relationship with red algae and highlighting its role as a structurally and functionally distinct fouling substrate.

Tunicates constituted a major component of the assemblage and clustered into several well-resolved clades, including colonial ascidians (*Distaplia bermudensis*, *Botrylloides niger*), solitary ascidians (*Microcosmus squamiger*, *Ciona intestinalis*), and the bioactive species *Ecteinascidia turbinata*. Additional invertebrate taxa were distributed across distinct phylogenetic branches, including the hydrozoan *Kirchenpaueria pinnata*, the bryozoan *Pectinella magnifica*, the polychaetae *Branchioma luctuosum*, the mollusk *Patella caerulea*, the cnidarian *Actinia fragacea*, and the shipworm *Teredo navalis*. Molecular clustering was largely congruent with morphological identification.

### 3.2 Strains isolation and biochemical characterization

A total of 52 culturable bacterial isolates (FB1 to FB29 on TCBS medium and YF1 to YF23 on TSA medium) were isolated from biofilms and tissues associated with the fouling organisms. Most isolates were Gram-negative coccobacilli and bacilli. Catalase and oxidase tests indicated substantial physiological diversity, with oxidase-positive isolates representing a major fraction of the community, consistent with aerobic or facultatively aerobic metabolisms typical of early surface colonizers (Supplementary Table S1).

### 3.3 Molecular identification of bacterial isolates

**Figure 4:**
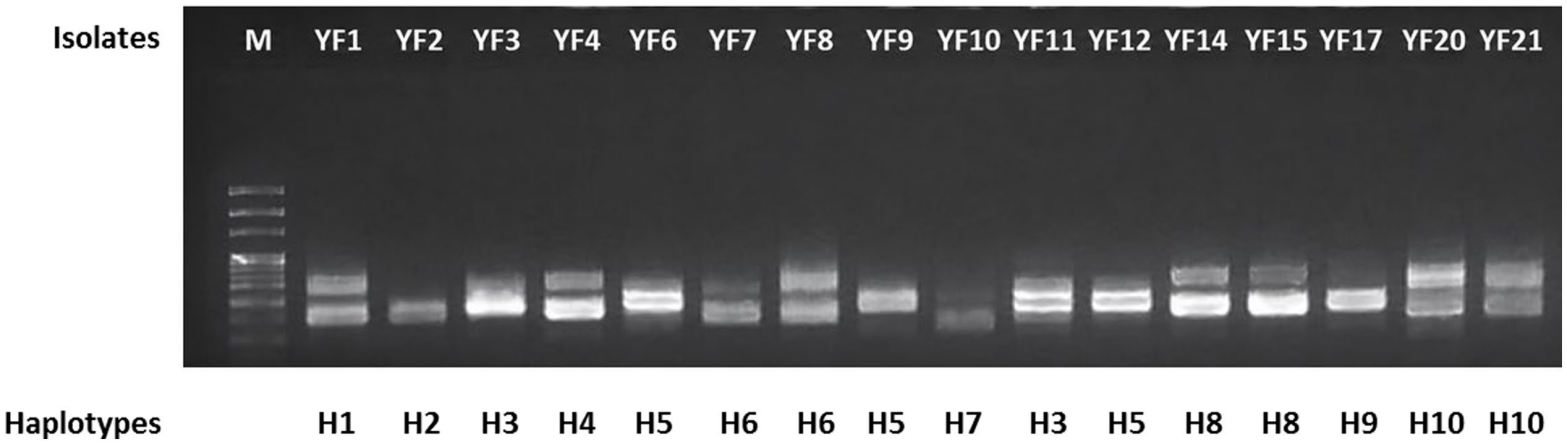
Representative ITS-PCR fingerprinting profiles of epibiotic bacterial isolates. Agarose gel electrophoresis (1% agarose) of amplified 16S–23S rRNA intergenic spacer region (ITS) fragments showing representative banding patterns obtained from selected isolates. Lanes marked “M” correspond to a 100 bp DNA ladder (Invitrogen™, USA). The different ITS banding profiles (haplotypes) were used to group the isolates prior to 16S rRNA gene sequencing for species-level identification.

ITS-PCR fingerprinting of the 52 bacterial isolates obtained from biofilm and biofouling samples collected along the Mediterranean coast of Tunisia generated reproducible profiles ranging from approximately 50 to 1,000 bp, allowing their classification into 27 distinct ITS haplotypes (Figure 4). One representative isolate per haplotype was selected for 16S rRNA gene sequencing to determine taxonomic affiliation at the species level (Supplementary Table S1). Sequence identity values between the obtained isolates and their closest matches in the BLAST database ranged from 99 to 100%, with a query coverage of 100% for all sequences (Table 2).

**Table 2:** Identification of bacterial isolates based on 16S rRNA gene sequence analysis.

Isolate ID	GenBank accession	Closest type strain	Reference strain accession	% Identity	Reported origin	Reference(s)
YF1_27f	PX928973	*Vibrio alginolyticus* NBRC 15630ᵀ	NR_122059.1	100%	Marine environments including seawater, sediments, fish, and shellfish	Gjerde and Böe (1981), Hörmansdorfer et al. (2000)
YF2_27f	PX928974	*Listeria innocua* ATCC 33090ᵀ	NR_116805.1	100%	Soil, water, vegetation and food-processing environments	Buchanan et al. (2017)
YF4_27f	PX928975	*Cobetia amphilecti* KMM 3880ᵀ	NR_113403.1	100%	Marine organisms such as sponges and seaweeds; marine environments	Romanenko et al. (2013), Noskova et al. (2021)
YF8_27f	PX928976	*Vibrio owensii* LMG 25443ᵀ	NR_117424.1	100%	Marine organisms including corals and shrimp	Ushijima et al. (2012)
YF14_27f	PX928977	*Pseudoalteromonas hodoensis* H7ᵀ	NR_126232.1	100%	Marine biofilms and seawater	Ivanova et al. (2004)
YF17_27f	PX928978	*Vreelandella alkaliphila* 18bAGᵀ	NR_042256.1	100%	Hypersaline and alkaline environments such as salt lakes	de la Haba et al. (2025)
YF19_27f	PX928979	*Cytobacillus firmus* NBRC 15306ᵀ	NR_112635.1	100%	Soil, alkaline environments and sediments	Patel and Gupta (2020)
YF20_27f	PX928980	*Halomonas aestuarii* Hb3ᵀ	NR_159155.1	100%	Estuarine and marine saline environments	Arahal et al. (2002)
FB1_27f	PX928981	*Vibrio owensii* LMG 25443ᵀ	NR_117424.1	100%	Coral and shrimp-associated marine bacterium	Ushijima et al. (2012)
FB4_27f	PX928982	*Photobacterium damselae* ATCC 33539ᵀ	NR_040831.1	100%	Marine fish, seawater and aquaculture systems	Rivas et al. (2013)
FB8_27f	PX928983	*Vibrio mediterranei* ATCC 43341ᵀ	NR_117896.1	100%	Marine sediments and coral-associated environments	Thompson et al. (2001), Pujalte and Garay (1986)
FB11_27f	PX928984	*Vibrio alginolyticus* ATCC 17749ᵀ	NR_118258.1	99.83%	Seawater and marine organisms	Molitoris et al. (1985)
FB13_27f	PX928985	*Staphylococcus equorum* PA231ᵀ	NR_027520.1	100%	Animal skin microbiota and fermented foods	Irlinger (2008)
FB15_27f	PX928986	*Pseudomonas yamanorum* 8H1ᵀ	NR_178342.1	100%	Soil environment	Arnau et al. (2015)
FB16_27f	PX928987	*Pseudomonas yamanorum* 8H1ᵀ	NR_178342.1	100%	Soil environment	Arnau et al. (2015)
FB19_27f	PX928988	*Staphylococcus equorum* PA231ᵀ	NR_027520.1	99.87%	Fermented food and dairy environment	Irlinger (2008)
FB20_27f	PX928989	*Pseudomonas yamanorum* 8H1ᵀ	NR_178342.1	100%	Soil environment	Arnau et al. (2015)
FB21_1492r	PX928990	*Vibrio barjaei* 3062ᵀ	NR_152641.1	99.66%	Marine environments and seawater	Dubert et al. (2016)
FB23_27f	PX928991	*Pseudomonas yamanorum* 8H1ᵀ	NR_178342.1	100%	Soil environment	Arnau et al. (2015)
FB24_27f	PX928992	*Pseudomonas yamanorum* 8H1ᵀ	NR_178342.1	99%	Soil environment	Arnau et al. (2015)
FB25_27f	PX928993	*Staphylococcus warneri* AW25ᵀ	NR_025922.1	100%	Human and animal skin microbiota	Ludwig et al. (2009)
FB26_27f	PX928994	*Pseudomonas yamanorum* 8H1ᵀ	NR_178342.1	100%	Soil environment	Arnau et al. (2015)
FB28_27f	PX928995	*Staphylococcus equorum* PA231ᵀ	NR_027520.1	100%	Fermented food and dairy environment	Irlinger (2008)

Percentage identity was determined by BLASTn comparison against the NCBI database. ᵀ indicates the type strain of the respective species.

**Figure 5:**
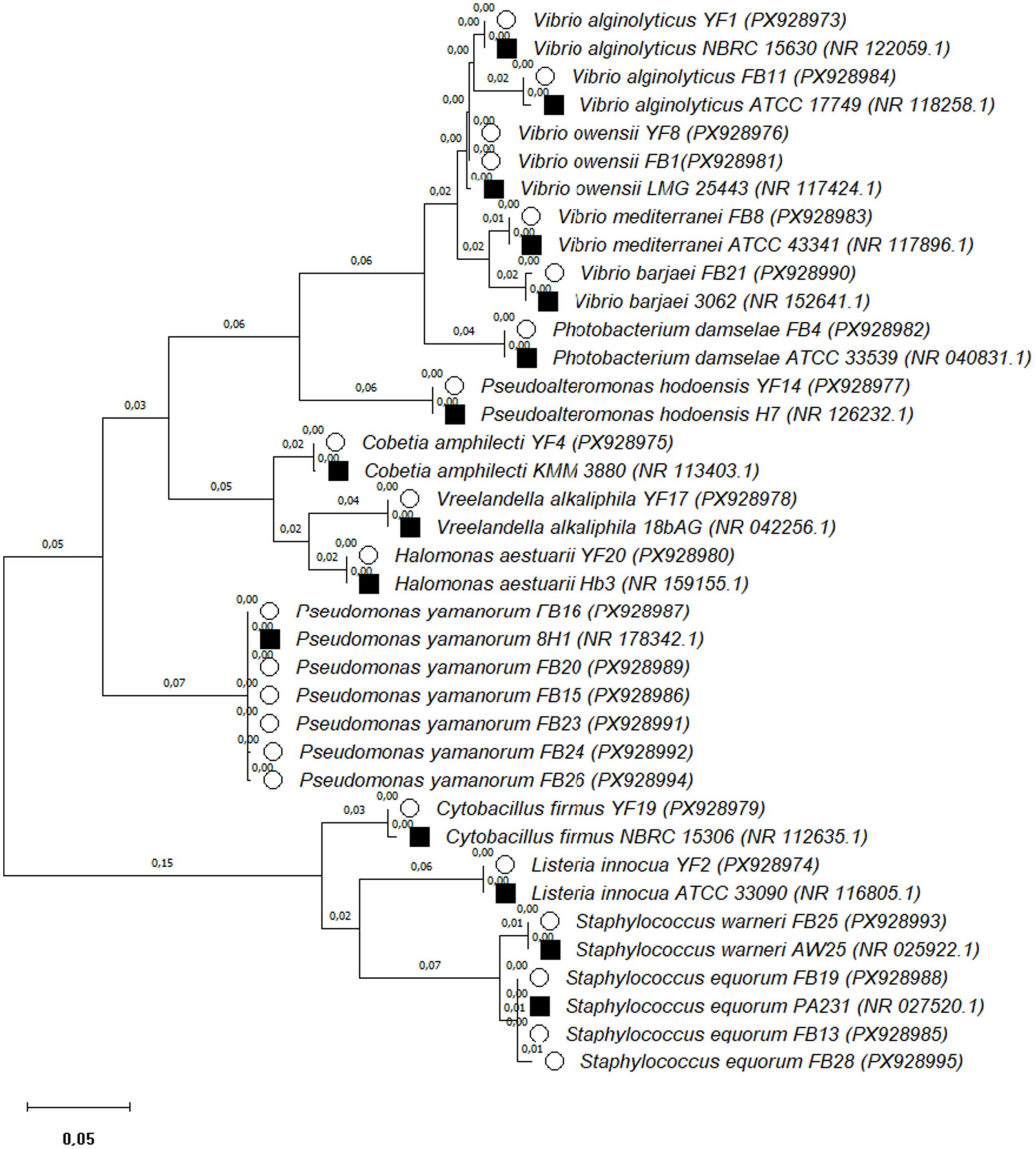
Maximum likelihood phylogenetic tree based on 16S rRNA gene sequences of bacterial isolates derived from marine invertebrate-associated biofilms. The tree was constructed using the Maximum Likelihood method and is drawn to scale, with branch lengths representing the number of substitutions per site (scale bar = 0.05). Numbers at nodes indicate bootstrap support values (1,000 replicates). *Listeria innocua* YF2 (PX928974) and *Listeria innocua* ATCC 33090 (NR_116805.1) were used as the outgroup to root the tree. Filled squares (■) represent the closest reference type strains retrieved from the NCBI database, while open circles (○) indicate the bacterial isolates obtained in this study. Accession numbers are given in parentheses. Isolates were recovered from the following host organisms: *Distaplia bermudensis* (YF1, YF4, YF17, FB1, FB4, FB8, FB15, FB16, FB21, FB26), *Kirchenpaueria pinnata* (YF19, YF20, FB11, FB24), *Chondrus crispus* (YF14, FB13, FB19), *Corallina caespitosa* (YF2, FB28), *Microcosmus squamiger* (YF8, FB25), *Ecteinascidia turbinata* (FB20), *Teredo navalis* (FB23), and *Pectinella magnifica* (FB8).

Phylogenetic analysis of the 16S rRNA gene sequences confirmed the taxonomic assignments and revealed distinct clades mainly within the Gammaproteobacteria (Figure 5), with *Vibrio* spp. and *Pseudomonas yamanorum* forming well-supported clusters, whereas Gram-positive taxa clustered separately and were less represented.

The culturable epibiotic bacterial community associated with Mediterranean biofilms and biofouling assemblages along the Tunisian coast was dominated by Gammaproteobacteria. Identified taxa included *Vibrio alginolyticus*, *Photobacterium damselae*, *Vibrio mediterranei* and *Vibrio barjaei*, as well as members of the genera *Pseudoalteromonas*, *Halomonas* and *Cobetia* (*Pseudoalteromonas hodoensis*, *Halomonas aestuarii* and *Cobetia amphilecti*). Gram-positive bacteria such as *Staphylococcus equorum*, *Staphylococcus warneri* and *Listeria innocua* were also detected.

Isolates were recovered from multiple biofouling hosts, including macroalgae (*Chondrus crispus*, *Corallina caespitosa*, *Amphiroa rigida*, *Amphiroa beauvoisii* and *Sargassum muticum*) and marine invertebrates, particularly tunicates (*Distaplia bermudensis*, *Microcosmus squamiger*, *Ecteinascidia turbinata*, *Botrylloides niger* and *Ciona intestinalis*) (Supplementary Table S1).

### 3.4 Antimicrobial activity of epibiotic culturable bacteria

**Figure 6:**
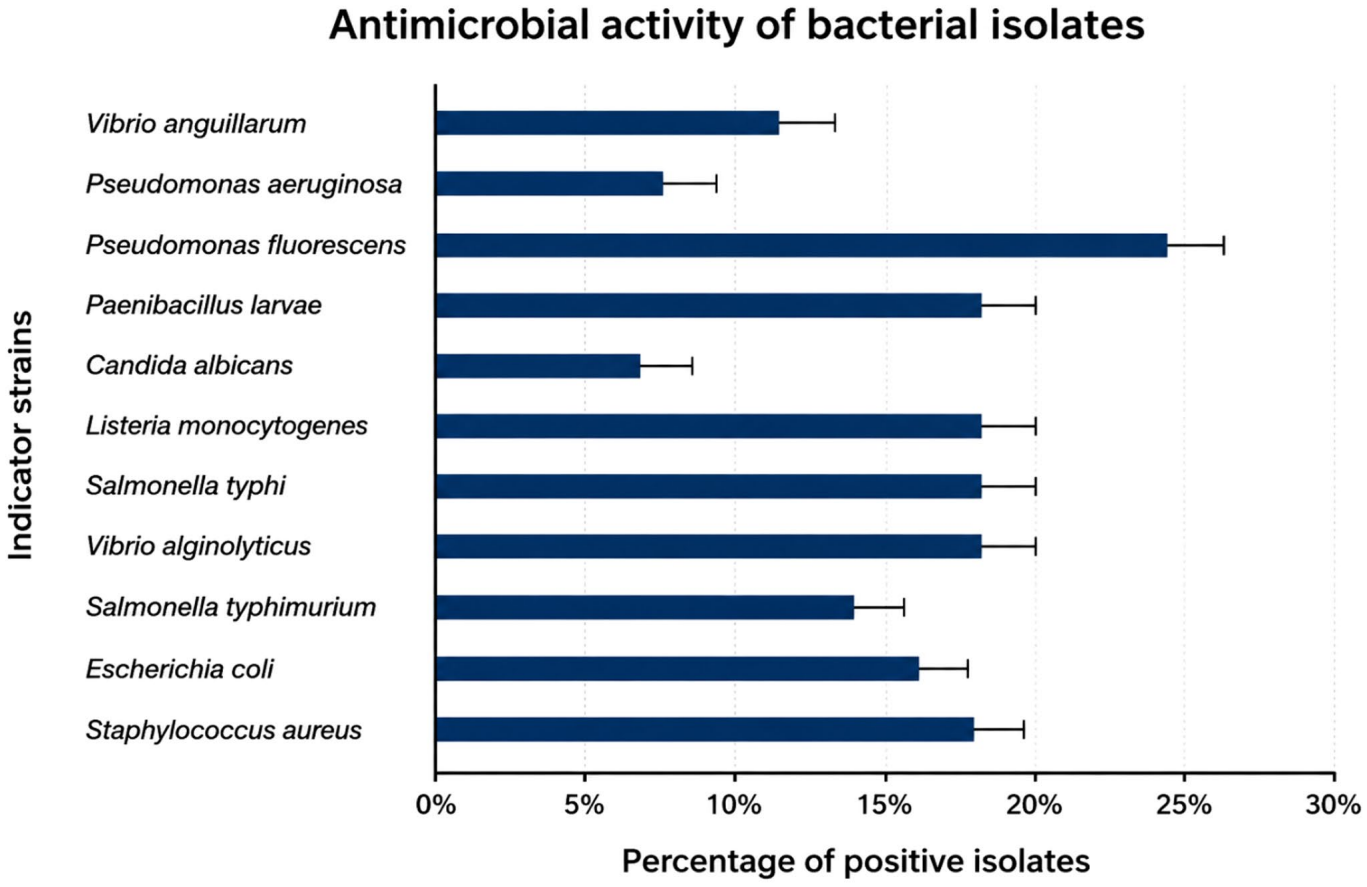
Antimicrobial activity spectrum of epibiotic isolates. Stacked bar chart showing the percentage of bacterial isolates (*n* = 52) that exhibited inhibitory activity against each target pathogen. Indicator strains included *Vibrio anguillarum* ATCC 12964 T, *Pseudomonas aeruginosa* ATCC 27853, *Pseudomonas fluorescens* AH2, *Paenibacillus larvae*, *Candida albicans* ATCC 10231, *Listeria monocytogenes* ATCC 7644, *Salmonella* spp., *Salmonella typhi* C52, *Vibrio alginolyticus* ATCC 17749 T, *Escherichia coli* ATCC 14948, and *Staphylococcus aureus* ATCC 25923. Antimicrobial activity was determined using the agar spot diffusion assay, and values represent the percentage of isolates producing visible inhibition zones against each target strain.

Antimicrobial activity assays revealed that a substantial proportion of the bacterial isolates exhibited inhibitory effects against the tested indicator microorganisms (Figure 6). The highest inhibition frequency was observed against *Pseudomonas fluorescens* (approximately 24%). Moderate inhibitory activity was recorded against *Paenibacillus larvae*, *Listeria monocytogenes*, *Salmonella typhi*, *Vibrio alginolyticus*, *Escherichia coli*, and *Staphylococcus aureus*, with inhibition percentages generally ranging between 15 and 18%. Lower inhibition frequencies were detected against *Salmonella typhimurium* (≈13%) and *Vibrio anguillarum* (≈11%). In contrast, *Pseudomonas aeruginosa* and *Candida albicans* were the least susceptible indicator strains, showing inhibition rates below 10%.

### 3.5 Enzymatic activities

**Figure 7:**
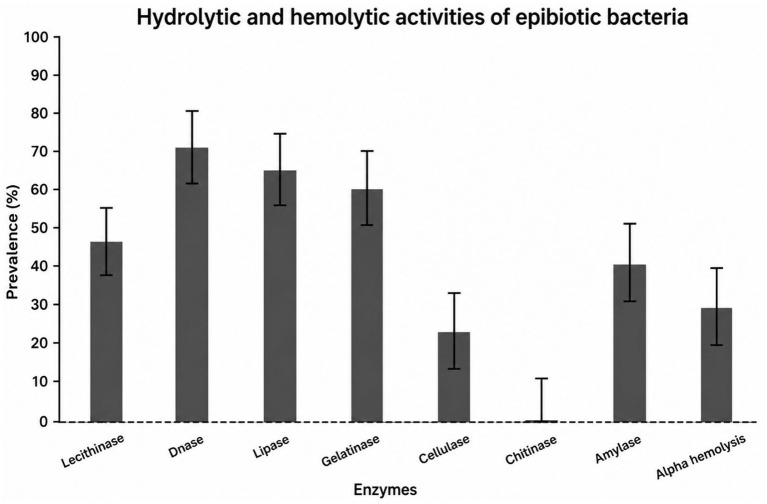
Hydrolytic enzyme profiles of epibiotic bacterial isolates. Bar chart showing the percentage of isolates (*n* = 52) positive for each enzymatic assay: lecithinase, DNase, lipase, gelatinase, cellulase, amylase, and hemolysis. Chitinase activity was not detected.

The enzymatic screening indicated a pronounced hydrolytic potential among the isolates (Figure 7). DNase activity was the most frequent, detected in 71.2% of isolates, demonstrating strong extracellular nucleic acid degradation capabilities. Lipase (65.4%) and gelatinase (59.6%) activities were also highly prevalent, reflecting efficient lipid and protein hydrolysis. The enzymatic activities of lecithinase, amylase, *α*-hemolysis and cellulase were moderately produced (respectively rates: 46.2, 42.3, 28.8 and 23.1% of isolates), while chitinase activity was absent.

### 3.6 Antibiotic susceptibility patterns

**Figure 8:**
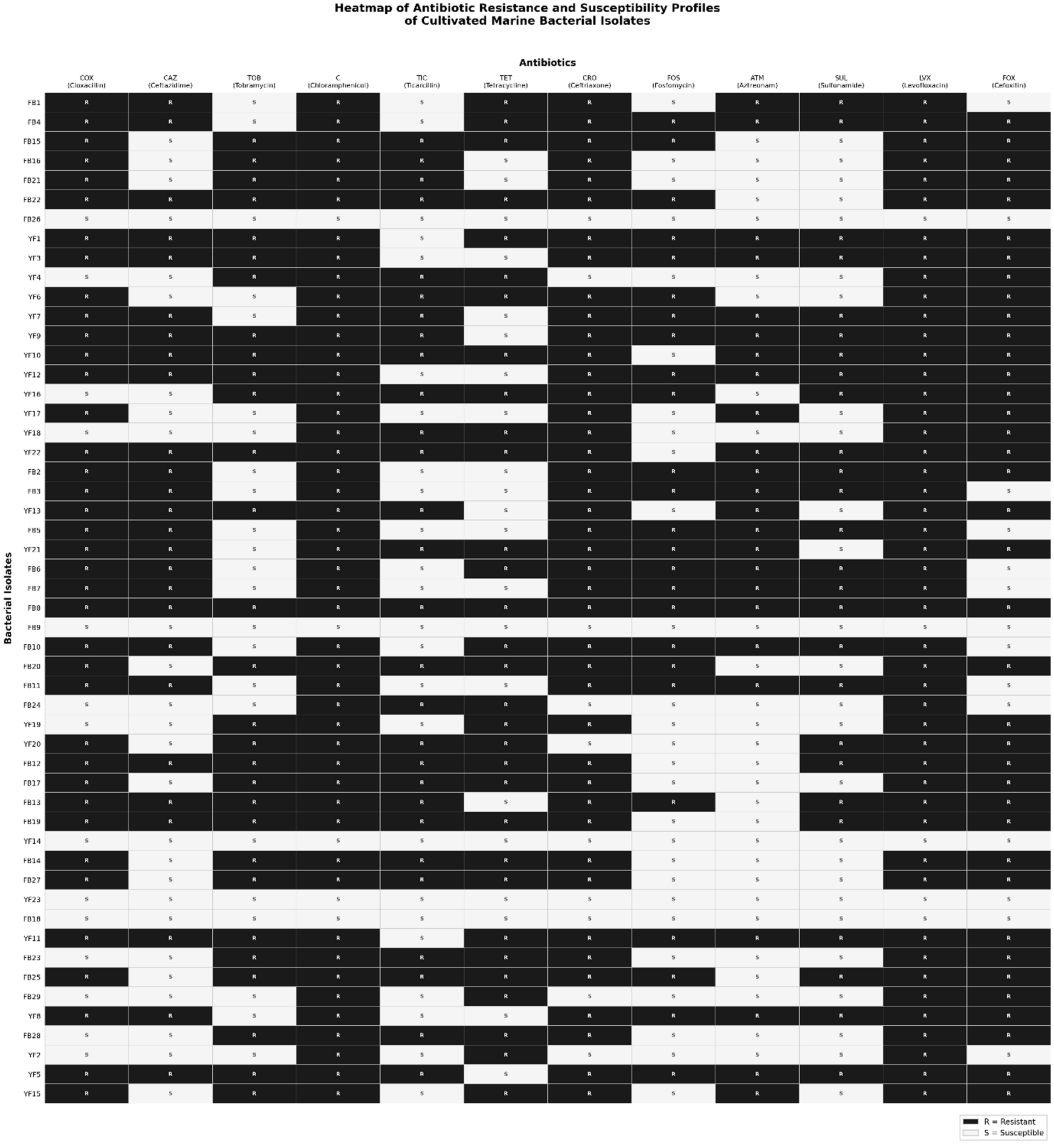
Heatmap of antibiotic resistance and susceptibility profiles of cultivated marine bacterial isolates. Each cell represents the susceptibility category of a given isolate against a tested antibiotic, classified as resistant (R), or susceptible (S) according to EUCAST breakpoint criteria. Antibiotics tested: ATM (aztreonam), C (chloramphenicol), CRO (ceftriaxone), CAZ (ceftazidime), COX (cloxacillin), FOS (fosfomycin), FOX (cefoxitin), LVX (levofloxacin), SUL (sulfonamide), TET (tetracycline), TIC (ticarcillin), and TOB (tobramycin).

**Figure 9:**
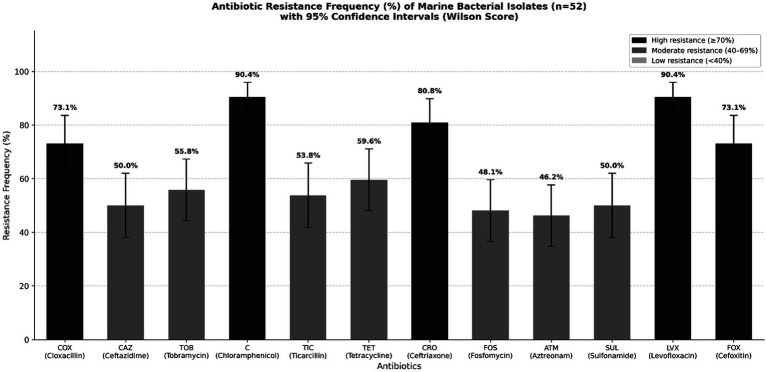
Antibiotic resistance frequency (%) of cultivated marine bacterial isolates (*n* = 52) with 95% confidence intervals (Wilson score method). Bar chart displaying the proportion of resistant isolates for each tested antibiotic, classified as resistant (R) according to EUCAST breakpoint criteria. Bars are colour-coded by resistance frequency: high (≥70%, black), moderate (40–69%, dark grey), and low (<40%, light grey). Antibiotics tested: COX (cloxacillin), CAZ (ceftazidime), TOB (tobramycin), C (chloramphenicol), TIC (ticarcillin), TET (tetracycline), CRO (ceftriaxone), FOS (fosfomycin), ATM (aztreonam), SUL (sulfonamide), LVX (levofloxacin), and FOX (cefoxitin).

Antibiotic susceptibility testing revealed heterogeneous responses to the tested antibiotic panel (Figures 8, 9). Large inhibition zones were generally observed for *β*-lactams such as ceftriaxone and aztreonam, indicating broad susceptibility, whereas reduced inhibition zones for tetracycline and tobramycin suggested partial or intrinsic resistance in several isolates. Based on EUCAST interpretative criteria, 34.6% of the isolates were resistant to at least one antibiotic, and 15.4% exhibited multidrug resistance (MDR). These results reflect only the culturable fraction of the bacterial community.

### 3.7 Statistical assessment of diversity, enzymatic activity, and antibiotic resistance

Bacterial morphotype diversity among the 51 isolates was estimated using the Shannon–Wiener diversity index (H′), the Simpson diversity index (D), and the Pielou evenness index (E), calculated from the relative abundance of the five identified morphotypes. The Shannon index yielded H′ = 1.160, the Simpson index D = 0.618, and the Pielou evenness index E = 0.720, indicating moderate diversity with relatively uneven distribution across morphotypes, dominated by coccobacilli (45.0%) and cocci (41.2%) (Table 3).

**Table 3:** Calculation of Shannon (H′) and Simpson (D) diversity indices for bacterial morphotypes.

Morphotype	ni (Number of isolates)	*N* (total)	pi = ni/*N*	ln(pi)	pi × ln(pi)	pi^2^
Coccobacille	23	51	0,450,000	-0,916,291	-0,412,331	0,202,500
Cocci	21	51	0,411,765	-0,887,303	-0,365,360	0,169,550
Bacille	5	51	0,098039	-2,322,388	-0,227,685	0,009612
Bacille en amas	1	51	0,019608	−3,931,826	−0,077095	0,000384
Bacille en chaine	1	51	0,019608	−3,931,826	−0,077095	0,000384
Total/Résultats	51		1,000000		−1,159,566	0,382,430
H′ (Shannon) = −Σ(pi×ln(pi))						1,159,566
D (Simpson) = 1 − *Σ*(pi^2^)						0,61,757
S (specific richness)						5
N (total population size)						51
E (Piélou evenness) = H′/ln(S)						0,72,047

H′ = −Σ(pᵢ × ln pᵢ); D = 1 − Σ(pᵢ^2^); E = H′ / ln(S); where pᵢ = nᵢ/N (relative frequency of morphotype i), S = number of morphotypes, n = total number of isolates.

The relationship between enzymatic activity and antibiotic resistance was assessed using Spearman’s rank correlation test (Table 4). Despite repeated attempts, antibiotic susceptibility testing could only be successfully performed on 47 of the 52 isolates, as five isolates failed to exhibit sufficient growth under the experimental conditions, rendering their resistance profiles unreliable for inclusion in the analysis. No significant correlation was detected in any group (Global: *r* = 0.104, *p* = 0.488; FB: *r* = 0.133, *p* = 0.519; YF: *r* = −0.008, *p* = 0.971), suggesting that enzymatic production and antibiotic resistance profiles are independent traits among the tested isolates.

**Table 4:** Spearman rank correlation between enzymatic activity score and antibiotic resistance score by isolate group.

Group	*n*	Σ[(Ri−R̄)(Si−S̄)]	Denominator	Spearman’s correlation coefficient (*r*)	*p*-value
Global	47	852,2,500	8,211,6,989	0,1,038	0,4,875
FB	26	—	—	0,1,326	0,5,186
YF	21	—	—	−0,0084	0,9,713

*r* = Σ[(Rᵢ − R̄)(Sᵢ − S̄)] / √[Σ(Rᵢ − R̄)^2^ × Σ(Sᵢ − S̄)^2^]; where Rᵢ = rank of enzymatic score, Sᵢ = rank of resistance score, R̄ and S̄ = mean ranks. Enzymatic score = number of positive activities among: lecithinase, DNase, lipase, gelatinase, cellulase, chitinase, and amylase (range 0–7). Resistance score = number of antibiotics to which the isolate was resistant out of 12 tested.

The relationship between enzymatic activity and antimicrobial activity against indicator strains was further evaluated by Spearman’s rank correlation on all 52 isolates (Table 5). No significant correlation was found in any group (Global: *r* = 0.231, *p* = 0.100; FB: *r* = −0.117, *p* = 0.544; YF: *r* = 0.162, *p* = 0.459), indicating that the capacity to produce hydrolytic enzymes does not predict antimicrobial inhibition potential in this collection.

**Table 5:** Spearman rank correlation between enzymatic activity and antimicrobial activity against indicator strains, by isolate group.

Group	*n*	Σ[(Rᵢ−R̄)(Sᵢ−S̄)]	√[Σ(Rᵢ−R̄)^2^ × Σ(Sᵢ−S̄)^2^]	*r* (Spearman)	*p*-value	Significance
Global	52	2316.50	10031.30	+0.2309	0.0995	ns (*p* > 0.05)
FB	29	−136.25	1160.82	−0.1174	0.5443	ns
YF	23	155.00	954.37	+0.1624	0.4591	ns

*r* = Σ[(Rᵢ − R̄)(Sᵢ − S̄)] / √[Σ(Rᵢ − R̄)^2^ × Σ(Sᵢ − S̄)^2^]; where Rᵢ = rank of enzymatic score, Sᵢ = rank of antimicrobial activity score, R̄ and S̄ = mean ranks. Enzymatic score = number of positive activities among lecithinase, DNase, lipase, gelatinase, cellulase, chitinase, and amylase (range 0–7). Antimicrobial activity score = number of indicator strains inhibited among *S. aureus*, *E. coli*, *S. typhimurium*, *V. alginolyticus*, Salmonella spp., Ps. aeruginosa, Listeria, *C. albicans*, *P. larvae*, Ps. fluorescens, and *V. anguillarum* (range 0–11). ns, not significant at α = 0.05.

The Multiple Antibiotic Resistance (MAR) index was calculated for each of the 47 successfully tested isolates using the formula MAR = a/b, where a represents the number of antibiotics to which an isolate was resistant and b represents the total number of antibiotics tested (*b* = 12). All 47 isolates exhibited MAR values exceeding the threshold of 0.2 (Global mean: 0.711, median: 0.750; FB mean: 0.696, median: 0.667; YF mean: 0.730, median: 0.750) (Table 6), indicating that all isolates originated from environments with high antibiotic selection pressure, as defined by Krumperman (1983).

**Table 6:** Multiple antibiotic resistance (MAR) index by isolate group (Krumperman, 1983).

Group	N tested	MAR min	MAR max	MAR average	MAR median	Isolats MAR > 0.2
Global	47	0,250	1,000	0,711	0,750	47
FB	26	0,333	1,000	0,696	0,667	26
YF	21	0,250	0,917	0,730	0,750	21

MAR, a/b; where a, number of antibiotics to which the isolate was resistant, b, total number of antibiotics tested (*b* = 12).

## 4 Discussion

### 4.1 A complex fouling assemblage: native foundations and invasive dominance

This study provides an integrated analysis of macrofaunal and microbial components within a Mediterranean biofouling community, revealing a highly interconnected system shaped by both native habitat-forming organisms and opportunistic or invasive species. Fouling assemblages serve as complex ecological interfaces where microbial biofilms interact with diverse macrofoulers, influencing biodiversity, ecosystem functioning, and biogeochemical processes, particularly in coastal environments under anthropogenic pressure (Salta et al., 2013; Dang and Lovell, 2016; Flemming et al., 2016; Dobretsov et al., 2019).

DNA barcoding revealed a structurally complex fouling community in the Marina in northern Tunisia, dominated by calcareous red algae (*Chondrus crispus, Corallina caespitosa, Amphiroa rigida,* and *Amphiroa beauvoisii*), which act as ecosystem engineers by providing stable three-dimensional surfaces that promote microbial attachment, biofilm maturation, and persistence. Other native fouling hosts included tunicates (*Distaplia bermudensis, Microcosmus squamiger, Ecteinascidia turbinata, Botrylloides niger,* and *Ciona intestinalis*), the hydrozoan *Kirchenpaueria pinnata*, the bryozoan *Pectinella magnifica*, the polychaeta *Branchioma luctuosum*, the gastropod *Patella caerulea*, the sea anemone *Actinia fragacea*, and the shipworm *Teredo navalis*. These structurally and taxonomically diverse substrates create a mosaic of microhabitats that likely shapes the composition, diversity, and functional dynamics of associated bacterial biofilms (Queirós et al., 2013; Pérez-Portela et al., 2018).

The presence of non-indigenous and opportunistic species, notably the invasive brown alga *Sargassum muticum* and the tunicates *Microcosmus squamiger* and *Botrylloides niger*, illustrates the ongoing biological invasions impacting Mediterranean coastal ecosystems (Rius et al., 2012; Zenetos et al., 2012). Invasive macrofoulers can modify surface characteristics and nutrient availability, influencing microbial colonization patterns, biofilm architecture, and interspecific microbial interactions (Dobretsov et al., 2019). The coexistence of native habitat-forming species with invasive taxa generates heterogeneous microhabitats, which likely promote epibiotic microbial diversity and dynamic biofilm–host relationships (Egan et al., 2013).

The environmental parameters measured during the sampling campaign indicate physicochemical conditions consistent with those commonly reported for Mediterranean coastal waters. The recorded temperature and salinity fall within typical regional ranges (Bethoux et al., 1999; Millot and Taupier-Letage, 2005), while the slightly alkaline pH is characteristic of seawater (Zeebe and Wolf-Gladrow, 2001). The dissolved oxygen concentration (4.52 mg/L) remains within a range compatible with aerobic marine environments, although below saturation levels (Garcia and Gordon, 1992). These parameters provide general environmental context for the studied system; however, as measurements were obtained at a single time point, caution is required when linking these conditions to observed biofilm composition or dynamics.

### 4.2 Molecular diversity and ecological implications

Molecular identification revealed a taxonomically and phylogenetically diverse epibiotic culturable bacterial community associated with biofilm and biofouling assemblages along the Mediterranean coast of Tunisia. This community was largely dominated by marine-adapted taxa commonly reported from surface-associated environments. This pattern may partly reflect the culture conditions used, which favored marine and salt-tolerant bacteria (Egan et al., 2013). Several *Vibrio* species identified here, including *Vibrio alginolyticus* and *Vibrio mediterranei*, have been previously documented in Mediterranean coastal waters and along the Tunisian shoreline in different marine matrices, including seawater and aquaculture contexts, where they have been associated with adhesion and virulence traits relevant to biofilm formation (Zaafrane et al., 2022). *Photobacterium damselae*, also common in marine environments globally, has been isolated from fish and other marine organisms in Mediterranean waters and is recognized as a widespread member of marine epibiotic communities (Morick et al., 2023).

The predominance of *Vibrio* spp. and *Pseudomonas yamanorum*, repeatedly isolated across multiple fouling hosts, underscores the ecological success of these genera in Mediterranean biofilm-forming habitats. Members of these groups are known for their metabolic versatility, rapid growth rates, and enhanced capacity to adhere to biotic and abiotic surfaces, traits that confer competitive advantages in dynamic biofouling systems. However, while *Vibrio alginolyticus* is well documented in regional seawater and associated biofilm studies (Zaafrane et al., 2022), certain taxa identified in the present study, such as *Pseudomonas yamanorum*, have only sporadic reports in marine settings and remain poorly represented in the literature for the Mediterranean Sea and Tunisia, indicating a more rare or underreported regional occurrence.

The observed genetic heterogeneity supports the core–rare biosphere paradigm, in which a limited number of dominant taxa co-occur with a broader assemblage of low-abundance lineages potentially acting as a functional reservoir under variable environmental conditions (Sogin et al., 2006; Lynch and Neufeld, 2015). Within this framework, recurrent taxa such as *Vibrio alginolyticus*, *Vibrio owensii* and *Pseudomonas yamanorum* likely constitute core components of the epibiotic microbiota associated with Mediterranean biofouling communities. In contrast, less frequently detected taxa, including *Pseudoalteromonas hodoensis*, *Vreelandella alkaliphila*, *Halomonas aestuarii*, *Cobetia amphilecti*, and *Cytobacillus firmus*, appear to be conditionally rare in regional marine environments, with few or no direct reports documenting their presence specifically along the Mediterranean basin or in Tunisian coastal studies. Such taxa may thus represent underexplored members of the marine epibiotic pool rather than well-established regional inhabitants.

Gram-positive bacteria such as *Staphylococcus equorum*, *Staphylococcus warneri* and *Listeria innocua* were also identified (Ivanek et al., 2009). These taxa are generally associated with non-marine environments or opportunistic lifestyles and are not commonly reported in Mediterranean marine biofilm studies, further highlighting the heterogeneity of the biofouling microbiota and the presence of taxa that may be transient or introduced via allochthonous sources.

The repeated isolation of specific bacterial species from phylogenetically and morphologically distinct fouling hosts, including macroalgae and tunicates, suggests the existence of relatively stable yet flexible host-associated microbial assemblages along the Tunisian Mediterranean coastline. Such patterns indicate that bacterial colonization may be driven more by surface ecology and local microhabitats than by strict host specificity. Conversely, the sporadic occurrence of certain taxa across samples points to microhabitat selection or transient colonization events, consistent with biofilm dynamics observed in other marine systems where surface chemistry, host morphology, hydrodynamics and environmental gradients jointly shape community structure (Egan et al., 2013; Pollet et al., 2018).

Importantly, several taxa identified in this study have been rarely reported or remain poorly documented in epibiotic biofilms from the southern Mediterranean, particularly along North African coasts. This includes species such as *Vibrio owensii*, *Pseudomonas yamanorum*, *Vreelandella alkaliphila* and *Cytobacillus firmus*, supporting the notion that significant gaps remain in the characterization of bacterial diversity associated with fouling organisms in this region. This underrepresentation reinforces the importance of integrating culture-dependent and culture-independent approaches to more comprehensively characterize the ecological functions and biotechnological potential of epibiotic bacteria on living substrates in Mediterranean coastal ecosystems (Quero et al., 2017).

### 4.3 Antimicrobial activity: ecological competition and biotechnological promise

A substantial proportion of the isolates exhibited antimicrobial activity, supporting the notion that chemical interference is a major driver of microbial competition in surface-associated communities (Long and Azam, 2001; Hibbing et al., 2010; Moradali et al., 2017). In biofilms, antimicrobial production constitutes an effective strategy to secure space and resources, particularly under conditions of high cell density and limited surface availability. This competitive behavior is closely linked to the biofilm lifestyle of opportunistic bacteria such as *Pseudomonas* spp., which display remarkable adaptive capacity and metabolic flexibility (Holmström and Kjelleberg, 1999; Moradali et al., 2017).

The reduced susceptibility observed for *Pseudomonas aeruginosa* aligns with previous reports suggesting the intrinsic tolerance of *P. aeruginosa* biofilms to antimicrobial agents. This tolerance arises from a combination of reduced penetration, physiological heterogeneity, and stress-induced adaptive responses within biofilm populations (Ciofu and Tolker-Nielsen, 2019). While this intrinsic resilience may limit the immediate effectiveness of certain antimicrobial compounds, it also underscores the ecological relevance of antimicrobial production as a selective force structuring biofilm communities. From an applied perspective, epibiotic biofilms remain promising reservoirs of novel bioactive metabolites with potential applications in antifouling and alternative antimicrobial strategies.

### 4.4 Hydrolytic enzyme production: a keystone trait for biofilm success

The high frequency of hydrolytic enzyme production observed among epibiotic culturable bacteria underscore the importance of extracellular enzymatic activity as a keystone functional trait in marine biofilms (Flemming and Wingender, 2010; Dang and Lovell, 2016). Enzymes such as DNases, lipases, and proteases play a crucial role in degrading extracellular polymeric substances and complex organic matter, thereby facilitating nutrient acquisition, biofilm remodeling, and competitive fitness within densely colonized surfaces (Dobretsov et al., 2019).

Such enzymatic versatility is characteristic of surface-associated bacteria capable of long-term persistence in biofilms. Biofilm-forming bacteria adopt adaptive strategies that enable them to exploit diverse nutrient sources while maintaining structural integrity and resistance to environmental stressors (Moradali et al., 2017). In fouling environments, hydrolytic activity likely contributes to the functional integration of microbial communities with their macrofouling hosts by mediating organic matter recycling and surface conditioning, ultimately influencing subsequent microbial and macro-organism settlement.

### 4.5 Antibiotic resistance: an environmental health concern in coastal waters

The detection of antibiotic resistance, including multidrug-resistant phenotypes, among epibiotic culturable isolates highlight the growing concern regarding antimicrobial resistance in coastal marine environments. Biofilm-associated bacteria are known to exhibit enhanced tolerance and resistance to antibiotics, owing to physiological heterogeneity, stress responses, and the protective nature of the biofilm matrix (Baquero et al., 2008., Martinez, 2009., Ciofu and Tolker-Nielsen, 2019).

Notably, *Pseudomonas* spp. represent key environmental reservoirs of resistance determinants. The increasing global dissemination of mobile carbapenemase genes in *P. aeruginosa* emphasizes the capacity of this species to acquire, maintain, and spread clinically relevant resistance genes (Yoon and Jeong, 2021). In biofouling environments, where high cell density and close physical proximity prevail, such resistance genes may persist and potentially be transferred among co-occurring bacteria, positioning biofilms as hotspots for antimicrobial resistance dissemination (Balcázar et al., 2015).

The coexistence of antimicrobial-producing bacteria and resistant strains within the same biofilm reflects a complex ecological balance between competition and persistence (Watts et al., 2017). However, given that this study was based on a single sampling campaign and did not include non-impacted reference sites, it is not possible to distinguish between baseline and anthropogenically influenced levels of antimicrobial resistance. Previous studies suggest that coastal environments subjected to anthropogenic inputs, such as wastewater discharge and urban runoff, may exhibit elevated levels of antibiotic residues and resistant bacteria (Zhang et al., 2019). In this context, the present findings should be interpreted as a preliminary indication of the presence of resistance traits, rather than a definitive assessment of environmental impact.

### 4.6 Functional traits and resistance patterns of culturable biofilm-associated bacteria

The moderate morphotype diversity observed among the 51 culturable epibiotic isolates (Shannon H′ = 1.160; Simpson D = 0.618; Pielou E = 0.720), dominated by coccobacilli (45.0%) and cocci (41.2%), reflects the structural heterogeneity within the cultivable fraction of the bacterial community associated with marine invertebrate biofilms. The relatively uneven distribution across morphotypes suggests that specific cell morphologies may confer selective advantages within biofilm microenvironments, potentially linked to surface attachment or resistance to environmental stressors. Spearman’s rank correlation analysis revealed no significant relationship between enzymatic activity and antibiotic resistance (Global: ***r* =** 0.104, *p* = 0.488), nor between enzymatic activity and antimicrobial inhibition potential (Global: ***r* =** 0.231, *p* = 0.100), in any of the tested groups. The absence of significant correlations between enzymatic activity, antibiotic resistance, and antimicrobial inhibition potential suggests that these traits are independently distributed across isolates, reflecting the broad and diverse functional repertoire of the culturable epibiotic community. This functional independence further underscores the ecological richness of marine invertebrate-associated biofilms, where multiple adaptive strategies may coexist without being mechanistically linked. Nevertheless, the uniformly high MAR index values recorded across all 47 successfully tested isolates (Global mean: 0.711; FB mean: 0.696; YF mean: 0.730), all exceeding the threshold of 0.2 defined by Krumperman (1983), indicate that these bacteria originated from environments subjected to sustained antibiotic selection pressure, consistent with the complex chemical ecology of marine invertebrate-associated biofilms. However, given the culture-dependent nature of this study and the absence of molecular approaches such as amplicon sequencing, these findings do not capture the full diversity of the microbial community, as widely reported in microbial ecology studies (Amann et al., 1995; Staley and Konopka, 1985; Lloyd et al., 2018; Martiny, 2019). Altogether, these results highlight the functional potential of culturable bacteria associated with marine invertebrate biofilms, while emphasizing the need for complementary molecular analyses to more comprehensively characterize the overall microbial diversity and ecological dynamics of these communities.

### 4.7 Synthesis and perspectives

Overall, this study provides a preliminary overview of the culturable epibiotic bacteria associated with biofouling communities in the Marina in northern Tunisia, based on a single sampling campaign. The results illustrate the functional potential of these bacteria in terms of enzymatic activity, antimicrobial interactions, and antibiotic resistance profiles. Biofilms developing on marine invertebrates appear to host bacterial assemblages capable of expressing diverse metabolic and antagonistic traits.

At the same time, the detection of antibiotic resistance among several isolates suggests that these biofilm-associated bacteria may reflect environmental conditions influenced by anthropogenic inputs. However, given that this study relies on culture-dependent approaches, the findings represent only the culturable fraction of the microbial community and should be interpreted with caution.

Further investigations combining culture-independent methods, repeated sampling efforts, detailed chemical characterization of bioactive compounds, and targeted analysis of resistance determinants will be necessary to better understand the ecological significance and potential applications of epibiotic biofilms in coastal marine environments.

## 5 Conclusion

The present work represents an integrated assessment of biofouling communities in a southern Mediterranean marina, linking macrofaunal diversity with the functional traits of associated bacteria. The epibiotic culturable community demonstrated substantial enzymatic and antimicrobial potential, highlighting its promise as a source of natural products for antifouling or therapeutic applications. However, the high prevalence of antibiotic resistance, especially among clinically relevant genera, signals an important environmental health risk. These findings emphasize the need to view coastal biofilms as dual-nature reservoirs: of biotechnological innovation and of genetic resistance. Future research should focus on compound isolation and ecological monitoring to harness their potential while mitigating associated risks.

## Data Availability

The datasets presented in this study can be found in online repositories. The names of the repository/repositories and accession number(s) can be found in the article/Supplementary material.

## Footnotes

1
http://www.ncbi.nlm.nih.gov/BLAST
